# OReFiL: an online resource finder for life sciences

**DOI:** 10.1186/1471-2105-8-287

**Published:** 2007-08-06

**Authors:** Yasunori Yamamoto, Toshihisa Takagi

**Affiliations:** 1 Department of Computational Biology, University of Tokyo, Kashiwanoha, Kashiwa, Chiba, Japan

## Abstract

**Background:**

Many online resources for the life sciences have been developed and introduced in peer-reviewed papers recently, ranging from databases and web applications to data-analysis software. Some have been introduced in special journal issues or websites with a search function, but others remain scattered throughout the Internet and in the published literature. The searchable resources on these sites are collected and maintained manually and are therefore of higher quality than automatically updated sites, but also require more time and effort.

**Description:**

We developed an online resource search system called OReFiL to address these issues. We developed a crawler to gather all of the web pages whose URLs appear in MEDLINE abstracts and full-text papers on the BioMed Central open-access journals. The URLs were extracted using regular expressions and rules based on our heuristic knowledge. We then indexed the online resources to facilitate their retrieval and comparison by researchers. Because every online resource has at least one PubMed ID, we can easily acquire its summary with Medical Subject Headings (MeSH) terms and confirm its credibility through reference to the corresponding PubMed entry. In addition, because OReFiL automatically extracts URLs and updates the index, minimal time and effort is needed to maintain the system.

**Conclusion:**

We developed OReFiL, a search system for online life science resources, which is freely available. The system's distinctive features include the ability to return up-to-date query-relevant online resources introduced in peer-reviewed papers; the ability to search using free words, MeSH terms, or author names; easy verification of each hit following links to the corresponding PubMed entry or to papers citing the URL through the search systems of BioMed Central, Scirus, HighWire Press, or Google Scholar; and quick confirmation of the existence of an online resource web page.

## Background

Many life-science-related online resources have been developed and introduced in journals such as BMC Bioinformatics, Bioinformatics, or Nucleic Acids Research [1]. These resources include databases. Web applications, and data-analysis software. Although some have been collected on web pages [[2,3], etc] or in special journal issues (*e.g*., Nucleic Acids Research (NAR) Database Issues [4]), others remain scattered throughout the Internet and within a vast amount of literature [5].

Some sites provide a curated collection of life-science-related online resources with a search function [[5,6], etc]. Because these sites maintain the contents manually, their quality is higher than sites that update automatically, but require more time and effort to manage. Web-page search engines such as Google [7] or Yahoo [8] do not exclusively retrieve online resources and do not necessarily show the relationship between the web page of an online resource and its corresponding publication information, which would allow researchers to measure its credibility.

Published papers that introduce an online resource often include a URL that readers can use to access the resource. Some journals that publish papers of introducing online resources (*e.g*., BMC Bioinformatics or Bioinformatics) require that authors state the availability of the resources, and many authors provide their resources using Hypertext Transfer Protocol (HTTP) or File Transfer Protocol (FTP). Therefore, we assumed that it would be useful to link the web page of an online resource to its corresponding publication information and build a search system. For example, BMC Bioinformatics offers papers in the category "Software." On 17 January 2007, we downloaded 277 full-text papers from the ftp site; only 10 of these did not have an available URL (*i.e*., 95.6% had a URL). Bioinformatics offers the paper category "Application Note." Of the 219 papers published in this category during 2006 and 2007 (from Volume 22, Issue 1 (22;1) to Volume 23, Issue 3 (23;3)), only one did not have a URL (i.e 99.5% had a URL.).

We developed an online search system, called OReFiL, with which researchers can easily find online resources of interest. Because every online resource that is retrieved has at least one link to a PubMed ID, users can easily acquire its summary and MeSH terms and confirm its credibility through reference to the corresponding PubMed entry. Every online resource can be searched using MeSH terms as a query, increasing the chance of finding query-relevant online resources. Because some sites such as BioMed Central [9], Scirus [10], HighWire Press [11], and Google Scholar [12] provide a search system covering full-text papers, OReFiL also provides links to their search services along with the hit URL. This function allows users to easily search for papers that cite the hit URL. Although an ideal retrieval system would be based on the infrastructure of the "resourceome" (the full set of bioinformatics resources) such as ontology or a peer-review system for resources [13], MeSH and peer-reviewed papers are available now and can be used. Automated URL extraction, web-page fetching, and indexing of the MEDLINE entries (which are essentially the same as PubMed data and share entry IDs) and fetched web pages minimizes the time and effort required to update the contents.

The target users of this system are researchers lacking in-depth knowledge in a specific field who are looking for online resources or experts to enhance their knowledge in that field. OReFiL is freely available [14].

## Construction and Content

The search system was developed in three stages: URL extraction, crawling, and indexing.

### Extraction

We extracted all of the URL expressions from 16 120 074 MEDLINE abstracts (2007 MEDLINE/PubMed Baseline Database [15]) through pattern matching using regular expressions. We checked the validity of all of the extracted expressions by looking up each host name (*e.g*., orefil.dbcls.jp). An expression was considered a valid URL if its host name was DNS resolvable and could return the server's Internet Protocol (IP) address, which means that the host name of the extracted expression exists in the Internet. At this step, we do not care if the web page of a valid URL actually exists. Because there are many irregular URL expressions in MEDLINE abstracts, they cannot all be extracted comprehensively and precisely [1]. For example, some abstracts omit the expression http://, and some have extra characters inserted in the URLs. Accordingly, we codified several extraction rules using our heuristic knowledge and developed an extraction tool. We evaluated the tool on NAR Web Server Issues and Database Issues published in 2004, 2005, and 2006. The tool correctly extracted URLs from 882 of a possible 883 abstracts.

In addition to the MEDLINE abstracts, we also extracted all of the URLs from BMC full-text papers with a PubMed ID (18 238 papers in total). BMC full-text papers are in the Extensible Markup Language (xml) format, and every URL is tagged within the tags <url> and </url>, simplifying the extraction. In BMC full-text papers, we scanned only the title, abstract, and sections with "availability" or "implementation" in their title. Some of the extracted URLs were publisher sites or company home pages, and we built a "stop list" that excluded them from the OReFiL's search results [see Additional file 1].

### Crawling

We developed a crawler to obtain the web pages of all of the extracted URLs. Because some pages lack HTML title tags (*i*.*e*.,<title> and </title>) or have an empty title, our crawler parses a page and finds the text that appears to be the title. In addition, our crawler considers HTML frame tags (*i*.*e*., <frameset> and </frameset>, <frame> and </frame>); some pages use these tags, which means the page for a given URL may contain no valuable text. The crawler also automatically follows "moved" pages or those web pages with an HTML meta tag containing the attribute HTTP-EQUIV="Refresh". Sometimes these represent sites that changed their URL after publication, so fetching the obsolete URL produces only the latest URL. The crawler will also try to access temporarily unavailable pages several times at regular intervals.

### Indexing

We used an open-source package called Lemur Toolkit [16,17] to index the obtained data and provide a search function. The Toolkit provides a well-structured query language and supports flexible search operations. The indexer indexed MEDLINE entries (*i*.*e*., title, abstract, author names, and MeSH terms) and the fetched web pages. The indexer only indexed the corresponding MEDLINE entries when the URL refers to an ftp site.

The indexer separately distinguishes MeSH terms, author names, titles, abstracts, and fetched web pages. In this way, OReFiL provides a context-dependent search function. This context-dependent search allows users to change the importance of each search variable. As for MeSH terms, OReFiL treats them in three different ways reflecting their importance and the MeSH hierarchy. Importance of a MeSH term depends on whether it is a major topic descriptor or not in a MEDLINE entry. In MEDLINE, a MeSH term is a major topic descriptor if it is one of the main topics discussed in the paper [18]. The followings are the three ways of considering MeSH terms by the indexer:

1. considers all of the annotated MeSH terms equally,

2. considers annotated MeSH terms that are major topics of a paper only, and

3. considers MeSH terms that fit the condition 2 and their conceptual ancestors in the MeSH hierarchy.

MeSH terminology provides a consistent way to retrieve information that may use different terminology for the same concepts [19]. MeSH organizes its terms in a hierarchical structure, and consideration of the hierarchy is done by adding ancestors of a MeSH term to the index.

In fetched web pages, all of the HTML tags, comments, style data, and program codes were removed before indexing. The indexer uses the Porter's stemming algorithm to make an index. Words that appear highly frequently and are not meaningful to be included in the index (stop words) are removed. We built the stop-words list based on that of PubMed . We added 24 terms or expressions to the PubMed stop-words list such as "ten", "i.e.", etc. Our list consists of 152 words [see Additional file 2].

We used dummy text data for web pages that could not be fetched by the crawler to prevent them from gaining a high ranking in the index. In a language model used by Lemur Toolkit, documents that have a higher likelihood of containing a given query get higher ranks (query likelihood ranking). This means that entries with a MEDLINE entry that do not have a fetched web page tend to achieve higher ranks, although these entries should be ranked lower because their web pages probably cannot be accessed.

## Utility and Discussion

We next discuss the data indexed by OReFiL, its search function and interface, and its advantages and limitations.

### Indexed Data

**Figure 1:**
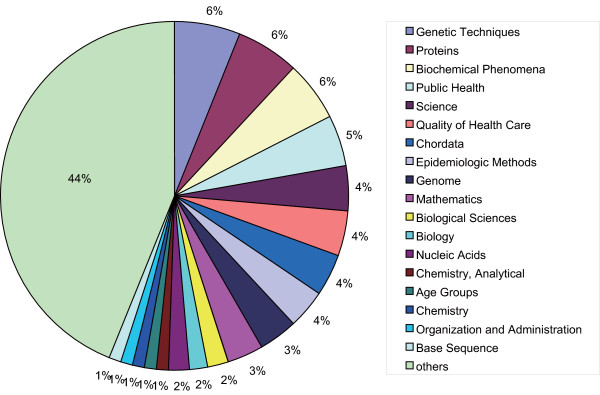
**MeSH term distribution**. MeSH term distribution at the second level of the hierarchy in those annotated to all the retrievable MEDLINE abstracts. Note that the following categories were excepted: "L" (Information Science). "V" (Publication Components), and "Z" (Geographic Locations).

The indexed data consisted of 7365 URLs and 8429 MEDLINE entries. Figure 1 shows the distribution of MeSH terms at the second level of the hierarchy. This indicates that the retrievable contents are well diversified and accordingly, a search with a MeSH term effectively narrows down resources.

### Search Function

OReFiL has language-model-based and Boolean-based search functions. Because OReFiL uses Indri in the Lemur Toolkit, users can use the rich and flexible query language to find query-relevant online resources. The simplest query consists of a word such as pathway. To find resources whose corresponding MEDLINE entry has a specific MeSH term, the user needs to add a query modifier that can be chosen from the following:

• *.mesh *(the specified MeSH term can be a major topic descriptor or not in the MEDLINE entry),

• *.noexp *(the specified MeSH term is a major topic descriptor in the MEDLINE entry), or

• *.majr *(in addition to the condition of .noexp, query expansion is on).

Search behaviors for these query modifiers correspond to the way of treating MeSH terms by the indexer. The system currently does not use MeSH Subheadings. An example of using a query modifier for MeSH is Proteins.mesh, in which OReFiL searches for resources for which the corresponding MEDLINE entries have the MeSH term "Proteins," but it does not consider whether the term is a major topic descriptor. An example of using the query expansion is Proteins.majr, in which OReFiL returns entries having the MeSH term "Proteins," and its descendants, such as "Caspase 9." Users can also search by an author's name using the modifier .auth such as smith.auth. The other modifiers are .atitle, .tiab, and .web. These are used to search for resources under the conditions that a specified term appears in their corresponding MEDLINE titles, MEDLINE titles and abstracts, and fetched web pages, respectively. To query with multiple words, the *#1 *operator is used. For example, in the query #1 (metabolic pathway), OReFiL searches for entries having the exact expression "metabolic pathway." For a Boolean "AND" search, the user needs to use the *#band *operator, such as #band(pathway apoptosis) to retrieve entries in which both "pathway" and "apoptosis" appear. Boolean-based and language-model-based searches are mutually exclusive, and the language-model-based ranking we briefly described above does not work when users conduct a Boolean-based search. Instead, users can use the *#filreq *operator such as #filreq(Caspases.noexp apoptosis) to search for resources relevant to apoptosis whose MEDLINE entries have the MeSH term of "Caspases," and the ranking is determined based on the language model. The operators can be combined such as #filreq(#band(pathway apoptosis) #l(Oxidative Stress).majr).

### Interface

**Figure 2:**
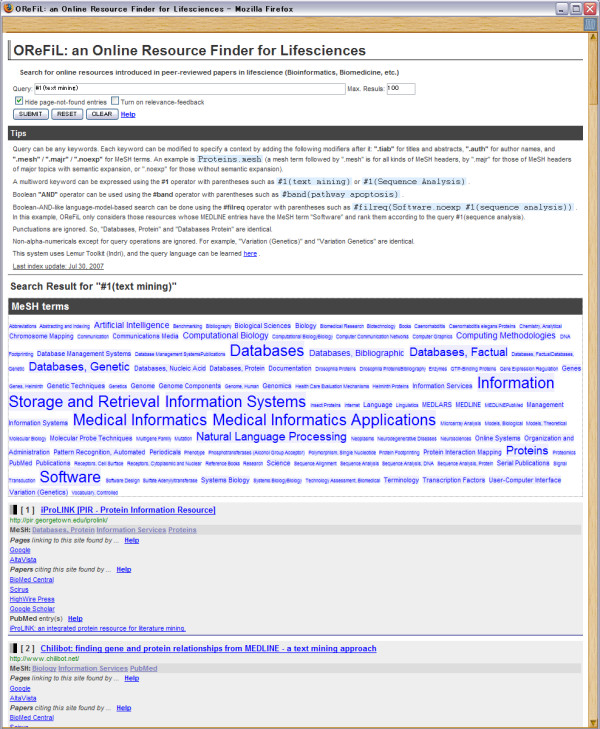
**Screen image of OReFiL**. This image shows the search result of the query *protein protein interaction. *MeSH terms annotated to the MEDLINE abstracts in the hit list and their conceptual ancestors in the MeSH hierarchy are displayed in the alphabetical order in the MeSH term box (encircled by a dotted line), and each font size reflects the frequency. MeSH terms also can be used to filter the result by narrowing down to those entries that have a specified MeSH term. Changing a query to narrow down is done by clicking a MeSH term in the box. Clicking a same MeSH term twice removes it from the query.

Figure 2 shows a screenshot of an OReFiL output. Its homepage is common to the search result pages (the area above the first horizontal line is displayed when you first access the site). In the MeSH terms box (encircled by a dotted line), MeSH terms (descriptors of major topics and their conceptual ancestors in the MeSH hierarchy) that are annotated to the MEDLINE abstracts included in the hit list are displayed in alphabetical order. Each font size reflects the frequency of the term in the hit list; terms that are more frequently annotated to the MEDLINE abstracts in the hit list are bigger and vice versa. OReFiL counts not only the annotated MeSH terms, but also their conceptual ancestors. MeSH terms also function as a filter to narrow down a search result. By clicking one of these, the browser automatically composes and appropriately fills in a required query. Users can easily remove a MeSH term from the query box by clicking the term. Users do not need to type in a complicated query to narrow down the results. After the user confirms the query box, clicking the "SUBMIT" button will produce the results.

The hit list appears below the MeSH term box. Each hit entry produces a grey box containing the following information:

• Title of a web page introduced in a peer-reviewed paper, with a link to the page and its URL,

• MeSH terms (descriptors of major topics),

• Links to popular web page search systems to search for web pages having a link to the hit web page,

• Links to major full-text paper journal search systems to search for papers citing the hit web page,

• Paper titles with a link to the corresponding PubMed entries.

We added the third and fourth items because they are useful to evaluate the reputation of the web page and acquire related knowledge. A hit entry has links to Google and AltaVista [20] to search for web pages that have a link to the web page of the entry. They provide the link: option and can retrieve web pages having a link to a given web page. For example, the search result for the query link:pubmed.gov is a list of web pages having a link to pubmed.gov. A hit entry also has links to (1) BioMed Central, (2) Scirus, (3) HighWire Press, and (4) Google Scholar to search for full-text papers in which the URL appears. Each link includes an appropriate query; therefore, users can get a search result after just clicking it.

Our crawler could not fetch some web pages. We currently classify these cases into the following three categories: (1) page not found, (2) network or server problem, and (3) other issues. In the first case, the server returns an HTTP status of 404; in the second case, the first digit of the status is 5 (5xx). Because the probability of a web page's removal is higher in the first than in the second case, we distinguish between the two. The 5xx code indicates a problem in the network or the server, so we determined that they needed to be distinguished from the other issues. Checking the "Hide page-not-found entries" box allows users to hide the entries in the first category from a hit list (it is checked as the initial setting).

### Advantages

First, general and very popular search systems such as Google, Yahoo, and AltaVista can be used to find online resources in the life science domain. However, it, is difficult to use these systems to find specific types of web pages. OReFiL only searches for web pages with URLs that have appeared in peer-reviewed journals in the domain.

Second, although PubMed only searches for peer-reviewed journal papers in the domain, its results are too broad for users who want to find online resources. In addition, PubMed does not search for related web pages or URLs not appearing in the paper abstracts. OReFiL currently covers MEDLINE entries and BMC full-text papers, and we are preparing to include other open-access journal papers, including the contents of NAR, Genome Research, and others at PubMed Central.

Third, BioMed Central maintains a catalog of more than 1100 database sites and provides subject-area-based browsing and search functions (BioMed Central Databases). However, they only focus on databases and do not provide links to peer-reviewed paper information.

Another domain-specific search system is maintained by the Health Sciences Library System at the University of Pittsburgh [5,21]. They provide a basic Boolean-based search function and a clustering-based browsing and narrowing down function. The clustering function is provided by a commercial software Vivisimo clustering engine [22] that automatically clusters documents based on their contents. Each online resource entry is manually curated, assuring good-quality results. However, this system takes more time and effort to update than does OReFiL. Other sites providing curated collections of resources also have this problem. In addition, OReFiL's MeSH-term-based search can be used to cluster resources.

To address these above issues, we developed an online resource search system in the life sciences that is up to date, covers a large number of resources in the domain, provides a link to peer-reviewed paper information, and has a flexible search function using an open-source toolkit. Because our crawler periodically accesses each URL, the existence of web pages can be checked.

### Limitations and points for improvement

Currently, OReFiL does not consider the context in which a URL appears; authors may be developers or users of a resource that they mentioned in the paper. Enabling URLs in both contexts to be searchable itself is not an issue, since it allows users to find resources that the developer has not introduced in an indexed peer-reviewed paper. The credibility of these resources can be verified by the number of PubMed entries in its entry or the impact factor of the journal in which the URL of the resource appears. However, such context information is beneficial to users, and we are planning to develop a method of identifying whether a paper introduces or uses a resource.

We also plan to develop an option for changing the ranking method. The current, system only evaluates the significance based on the language model; it is influenced by the distribution and frequency of terms in the target documents. It would also be desirable for OReFiL to be able to rank a query-relevant hit list by reputation or published dates.

The ability to display the part of a hit document (MEDLINE entry or fetched web page) in which a query term appears would also be useful to users because it is sometimes difficult to determine the relevance of the hit. We are developing a method to do this.

Our crawler currently cannot follow some "moved" pages. In some cases, a site has changed its URL several times, and each of the URLs is in MEDLINE abstracts. OReFiL identifies a resource as its corresponding URL; therefore, such a site will appear multiple times in the hit list that is generated. We need to fix this "URL synonym" issue.

**Figure 3:**
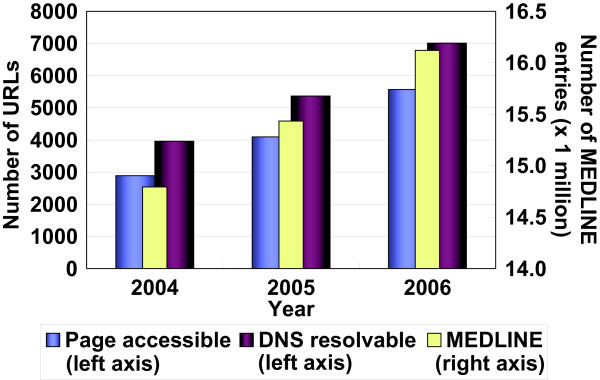
**Growth of online resources and MEDLINE**. The numbers of URLs appeared in MEDLINE abstracts. The number of the DNS-resolvable URLs is that of URLs whose server name can be resolvable. The number of the page-accessible URLs is that of URLs whose page can be accessed (the server returns the HTTP status code of 200). MEDLINE growth is added for reference.

Expanding OReFiL's coverage is a difficult task because many life science journals still do not allow open access to their full-text papers. Nevertheless, because both the number of URLs in MEDLINE abstracts (see Fig. 3 and [1]) and the number of journals allowing open access [23] are steadily increasing, OReFiL will become more useful. In addition, accepting user's submitted URLs and their corresponding PubMed IDs will improve OReFiL's coverage.

## Conclusion

We developed OReFiL, a search system for online life science resources that links related web pages to MEDLINE entries. It provides a flexible search using a language- and a Boolean- models, and enables users to search by MeSH terms using its conceptual hierarchy, author names, and free words. In addition, it offers an easy search refinement function. The index is updated automatically and thus requires minimal user effort to maintain.

## Availability and requirements

OReFiL can be freely available at .

## Abbreviations

• DNS: Domain Name System

• HTML: HyperText Markup Language

• MeSH: Medical Subject Headings

• URL: Uniform Resource Locator

## Authors' contributions

YY proposed and implemented the system and TT supervised the project and helped to draft the manuscript. All authors read and approved the final manuscript.

## Supplementary Material

Additional file 1 Stop-URL list. URL list to be removed from the index because of inappropriateness in terms of the purpose of searching for online resources. Click here for file

Additional file 2 Stop-word list. Word list to be removed from the index. Click here for file
