# Supplementary material for: OReFiL: an online resource finder for life sciences
Source: BMC Bioinformatics. 2007 Aug 6;8:287. doi: 10.1186/1471-2105-8-287 (PMC1976328; doi:10.1186/1471-2105-8-287)
Supplement: Additional file 1 — Stop-URL list. URL list to be removed from the index because of inappropriateness in terms of the purpose of searching for online resources. [file 1471-2105-8-287-S1.pdf]

Patterns for removing URLs from those to be indexed

m|/dx¥.(?:doi¥.)?org|i (Perl regular expression)

.oupjournals.org

.oxfordjournals.org

.psychonomic.org

circres.ahajournals.org

circresaha.org

interscience.wiley.com/jpages/

link.springer-ny.com

reprint

zhgz.chinajournal.net.cn

www.maik.ru

www.insp.mx

www.bjcancer.com

www.genome.org

www.rbmonline.com/Article/
