# Supplementary material for: OReFiL: an online resource finder for life sciences
Source: BMC Bioinformatics. 2007 Aug 6;8:287. doi: 10.1186/1471-2105-8-287 (PMC1976328; doi:10.1186/1471-2105-8-287)
Supplement: Additional file 2 — Stop-word list. Word list to be removed from the index. [file 1471-2105-8-287-S2.pdf]

Stop-words list

A
about, again, al., all, almost, also, although, always, among, an, and, another, any, are, as, at

B
be, because, been, before, being, between, both, but, by

C
can, could, did

D
do, does, done, due, during

E
e.g. , each, eight, either, enough, especially, et., et al., etc

F
five, for, found, four, from, further

H
had, has, have, having, here, how, however

I
i, i.e., if, in, into, is, it, its, itself

J
just

K
kg, km

M
made, mainly, make, may, mg, might, ml, mm, most, mostly, must

N
nearly, neither, nine, no, nor, not

O
obtained, of, often, on, one, or, our, overall

P
perhaps

Q
quite

R
rather, really, regarding

S
seem, seen, seven, several, should, show, showed, shown, shows, significantly, since, six, so, some, such

T
ten, than, that, the, their, theirs, them, then, there, therefore, these, they, this, those, three, through, thus, to, two

U
upon, use, used, using

V
various, very

W
was, we, were, what, when, where, whether, which, while, who, why, with, within, without
